# Plasmalogens Alter the Aggregation Rate of Transthyretin and Lower Toxicity of Transthyretin Fibrils

**DOI:** 10.1021/acs.jpclett.4c00868

**Published:** 2024-04-25

**Authors:** Jadon Sitton, Abid Ali, Luke Osborne, Aidan P. Holman, Axell Rodriguez, Dmitry Kurouski

**Affiliations:** † Department of Biochemistry and Biophysics, Texas A&M University, College Station, Texas 77843, United States; ‡ Department of Entomology, Texas A&M University, College Station, Texas 77843, United States; § Department of Biomedical Engineering, Texas A&M University, College Station, Texas 77843, United States

## Abstract

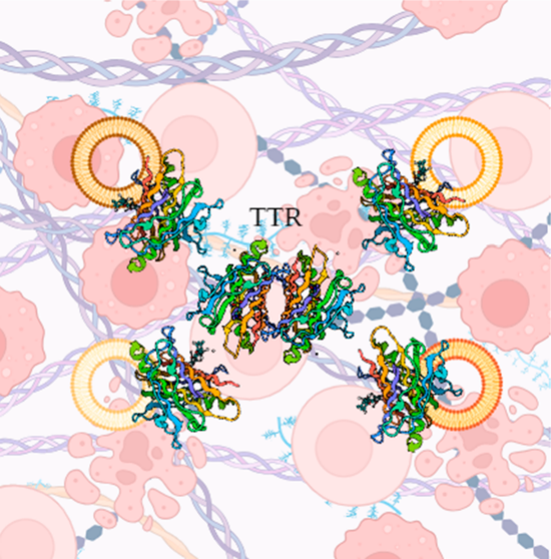

Heart tissue can experience a progressive accumulation of transthyretin (TTR), a small four subunit protein that transports holoretinol binding protein and thyroxine. This severe pathology is known as transthyretin amyloid cardiomyopathy. Numerous experimental studies indicated that the aggregation rate and toxicity of TTR fibrils could be altered by the presence of lipids; however, the role of plasmalogens in this process remains unknown. In this study, we investigate the effect of choline plasmalogens (CPs) with different lengths and saturations of fatty acids (FAs) on TTR aggregation. We found that CPs with saturated and unsaturated FAs strongly suppressed TTR aggregation. We also found that CPs with saturated FAs did not change the morphology of TTR fibrils; however, much thicker fibrillar species were formed in the presence of CPs with unsaturated FAs. Finally, we found that CPs with C16:0, C18:0, and C18:1 FAs substantially lowered the cytotoxicity of TTR fibrils that were formed in their presence.

Transthyretin amyloid cardiomyopathy is a severe disease which is caused by a progressive accumulation of transthyretin (TTR) in myocardium.^1−6^ TTR is a tetrameric protein that circulates through the body transporting retinol (vitamin A) and thyroxine.^7−11^ The Kelly group showed that TTR monomerization drastically lowers protein stability.^12−14^ As a result, β-sheet-rich monomers can aggregate forming amyloid oligomers and fibrils.^15,16^ Although there is very little if any information about structure of TTR oligomers, the structure of ex vivo extracted TTR fibrils was resolved by cryo-EM.^17^ It was found that, in TTR fibrils, two monomers were folded into a stable β-sheet structures that were stabilized by hydrogen bonding.^17^ These structures propagated micrometers in the direction that is perpendicular to β-strands. Similar experimental results were reported by Schmidt and co-workers for V30 M mutant of this protein.^18^

Recently reported results by Ali and co-workers demonstrated that the TTR aggregation rate could be altered by phospholipids.^19−21^ Furthermore, the change in the rate of TTR aggregation was directly dependent on the saturation of fatty acids (FAs) in the phospholipids. Specifically, unsaturated phosphatidic acid (PA) accelerated, whereas PA with saturated FA decelerated TTR aggregation.^20^ It was also demonstrated that the presence of both phosphatidylserine (PS) and PA lowered the toxicity of TTR fibrils formed in the presence of these phospholipids.^20,21^ The same findings were extended for FAs with different lengths and a degree of unsaturation.^19^ These results indicated that FAs and phospholipids could be used as therapeutic platforms to decrease the aggregation rate of TTR and lower the toxicity of TTR fibrils. These findings also demonstrate that progressive changes in the lipid profile of cell membranes present in the areas of TTR localization can trigger aggregation and accumulation. This, consequently, could cause the onset and progression of transthyretin amyloidosis.

Heart tissue has a high concentration of choline plasmalogens (CPs). These phospholipids have a unique vinyl ether linkage at the *sn*-1 site of glycerol.^22^ Although the actual physiological role of plasmalogens is unclear, numerous studies indicated that CPs could alter the rigidity of lipid bilayers. Thus, their localization at the sites of membrane fusion is expected.^22,23^ Extraordinary stability of the vinyl-ether linkage of CPs makes these molecules highly efficient in scavenging free radicals. Thus, CPs protect cells from reactive nitrogen and oxygen species.^22,24^ However, the role of CPs in the aggregation of amyloidogenic proteins is unclear.^19−21^ Recent studies from our group showed that CPs altered the aggregation rate of α-synuclein, a small membrane protein that is linked to Parkinson’s disease.^25^ However, no changes in the toxicity of fibrils formed in the presence and absence of CPs by α-synuclein were observed. Expanding upon this, we investigated the extent to which CPs composed of small lipid vesicles (SUVs) with different lengths and saturations of FAs (Scheme 1) could alter TTR aggregation. Using several biophysical techniques, we also determined the morphology and secondary structure of TTR fibrils grown in the presence of these CPs. Finally, we utilized rat N27 dopaminergic cells to examine toxicity of TTR fibrils that were grown under different experimental conditions.

**Scheme 1:**
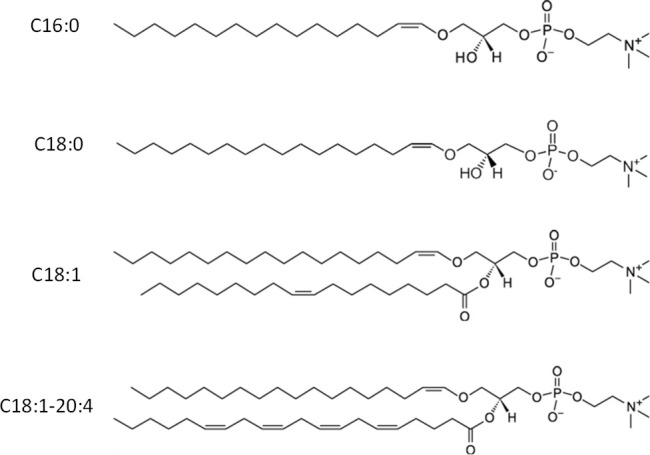
Molecular Structure of C16:0, C18:0, C18:1, and C18:1–C20:4 CPs

Thioflavin T (ThT) assay revealed that, at pH 3.0 and 37 °C, monomeric TTR aggregate exhibited a very short lag-phase (*t*_lag_ = 1.1 ± 0.98 h), Figure 1. We observed a significant delay in the lag-phase of TTR aggregation if both C16:0 and C18:0 (*t*_lag_ = 2.5 ± 0.3 and 2.6 ± 0.1 h, respectively) were present at equimolar concentrations with the protein. Even a greater delay in TTR aggregation was observed in the presence of CPs with unsaturated FAs. Specifically, in the presence of C18:1, TTR aggregated with *t*_lag_ = 7.6 ± 0.6 h, whereas in the presence of C18:1-C20:4, *t*_lag_ = 5.6 ± 0.0 h was observed. Thus, we can conclude that CPs strongly suppressed TTR aggregation. Furthermore, the effect of the suppression of TTR aggregation was directly dependent on the saturation of FAs in CPs.

**Figure 1:**
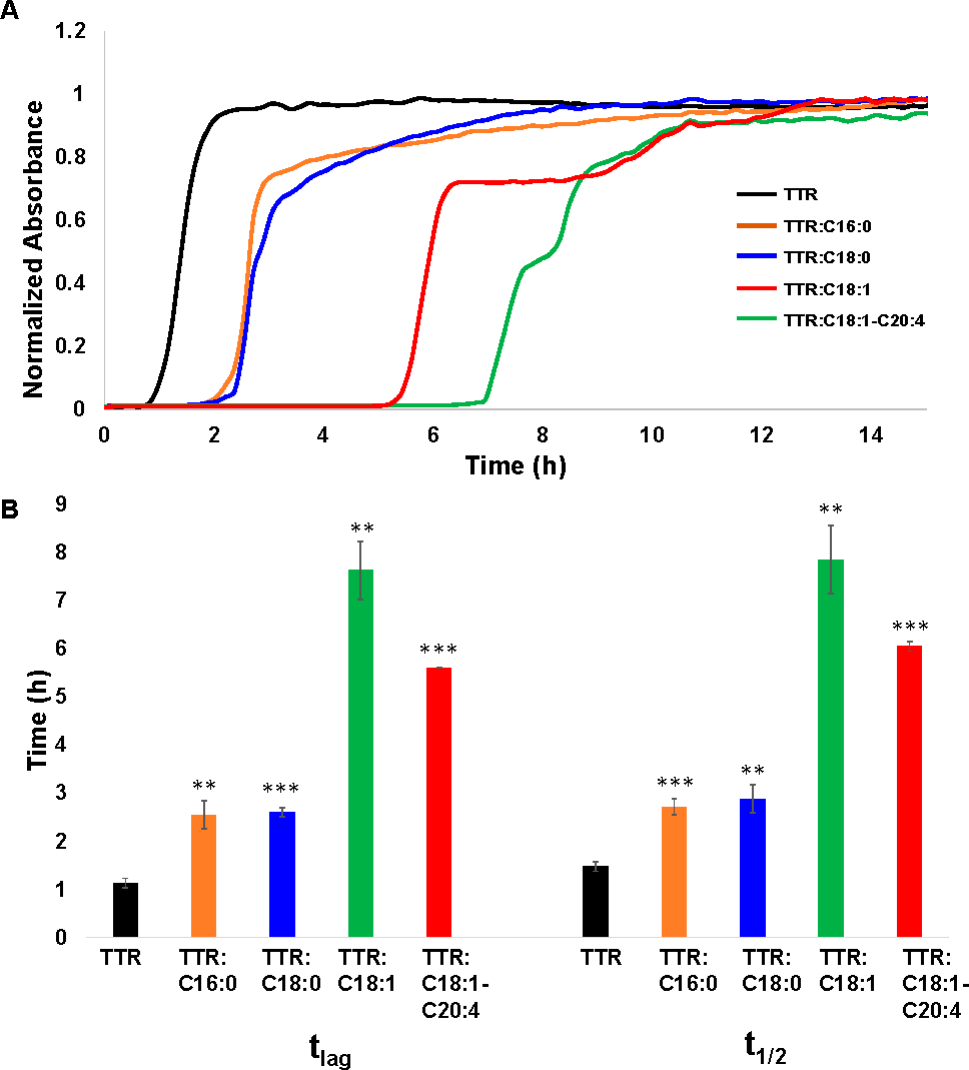
CPs alter the rate of TTR aggregation. Averaged (*n* = 3) ThT kinetics (A) and corresponding histograms (B) of TTR aggregation in the absence and presence of CPs. *p < 0.05, **p < 0.01, ***p < 0.001.

We also found that saturated CPs decelerated the rate of TTR aggregation from *t*_1/2_ = 1.5 ± 0.1 h (TTR) to *t*_1/2_ = 2.7 ± 0.2 h (TTR:C16:0) and *t*_1/2_ = 2.9 ± 0.3 h (TTR:C18:0). Even a greater effect was observed for the unsaturated CPs. Specifically, we found *t*_1/2_ equal to 7.9 ± 0.7 h in the presence of TTR:C18:1 and 6.0 ± 0.1 h in the presence of TTR:C18:1-C20:4. Thus, we can conclude that CPs also changed the aggregation rate of TTR if they were present at the stage of protein aggregation. Similar to the discussed above lag phase, we observed that CPs with unsaturated FAs exerted a greater effect on the suppression of TTR aggregation compared to CPs with saturated FAs.

Using atomic force microscopy (AFM), we investigated the morphology of TTR aggregates formed in the presence and absence of CPs, Figure 2.

**Figure 2:**
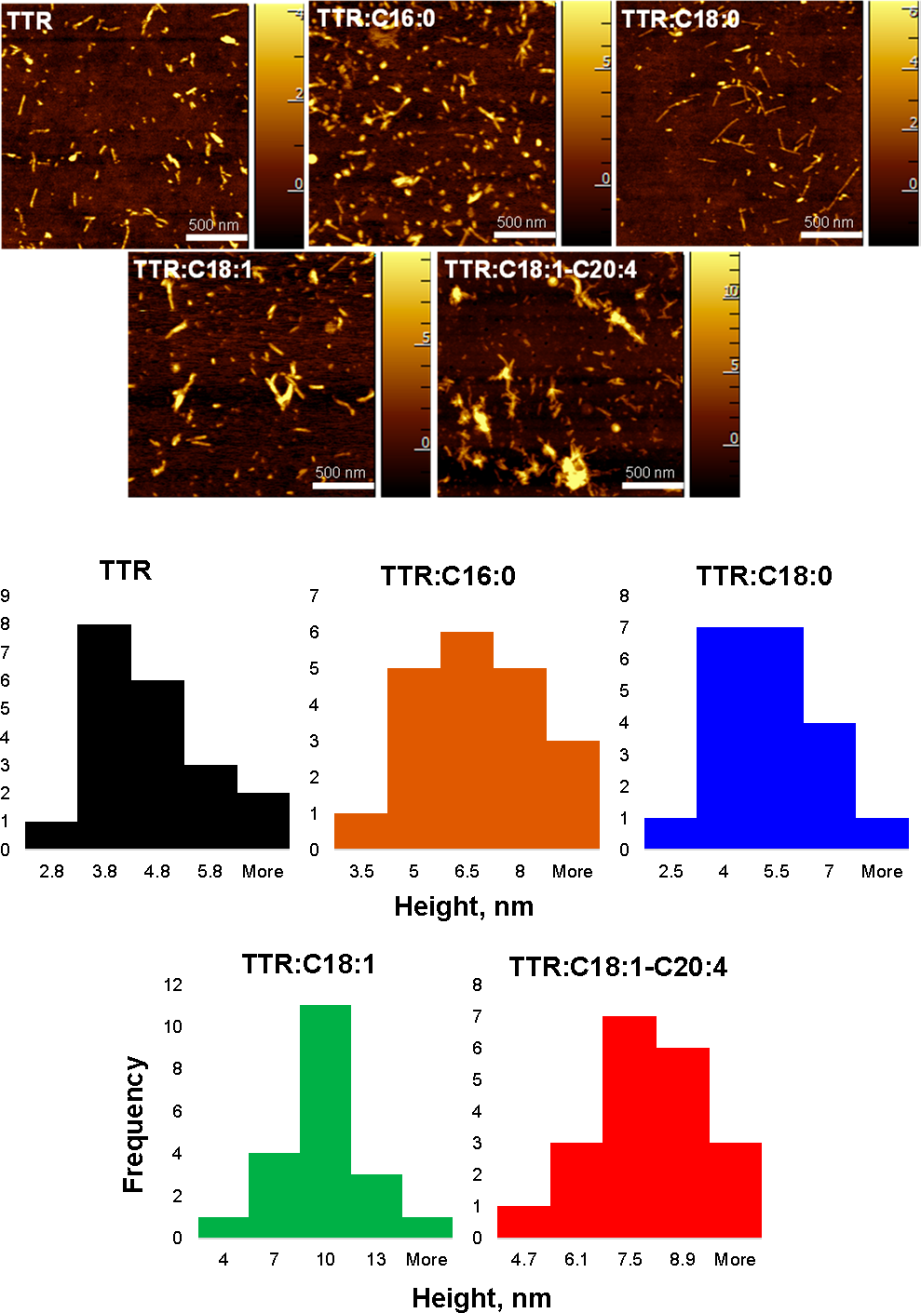
Morphological characterization of protein aggregates. AFM images (top) with corresponding histograms (bottom) of TTR aggregates formed in the presence and absence of CPs. AFM images were collected using an AIST-NT-HORIBA system (Edison, NJ) in tapping mode.

AFM revealed that, in the absence of CPs, TTR formed short 200–300 nm fibrils that were 2.8–6 nm in height. Morphologically similar fibrils were observed in TTR:C16:0 and TTR:C18:0. However, we found that their average height slightly increased from ∼4 nm (TTR) to ∼6.5 nm (TTR:C16:0) and 5 nm (TTR:C18:0), Figure 2. We also found that, in the presence of CPs with unsaturated FAs, TTR formed much thicker fibrils with average heights of 10 nm (TTR:C18:1) and 7.5 nm (TTR:C18:1-C20:4). Thus, we can conclude that CPs altered the topology of TTR fibrils that were formed in their presence. Furthermore, the change in the fibril morphology was much greater for CPs with unsaturated FAs compared with CPs with saturated FAs.

Next, we used infrared (IR) spectroscopy and circular dichroism (CD) to determine whether the presence of CPs altered the secondary structure of TTR aggregated, Figure 3.

**Figure 3:**
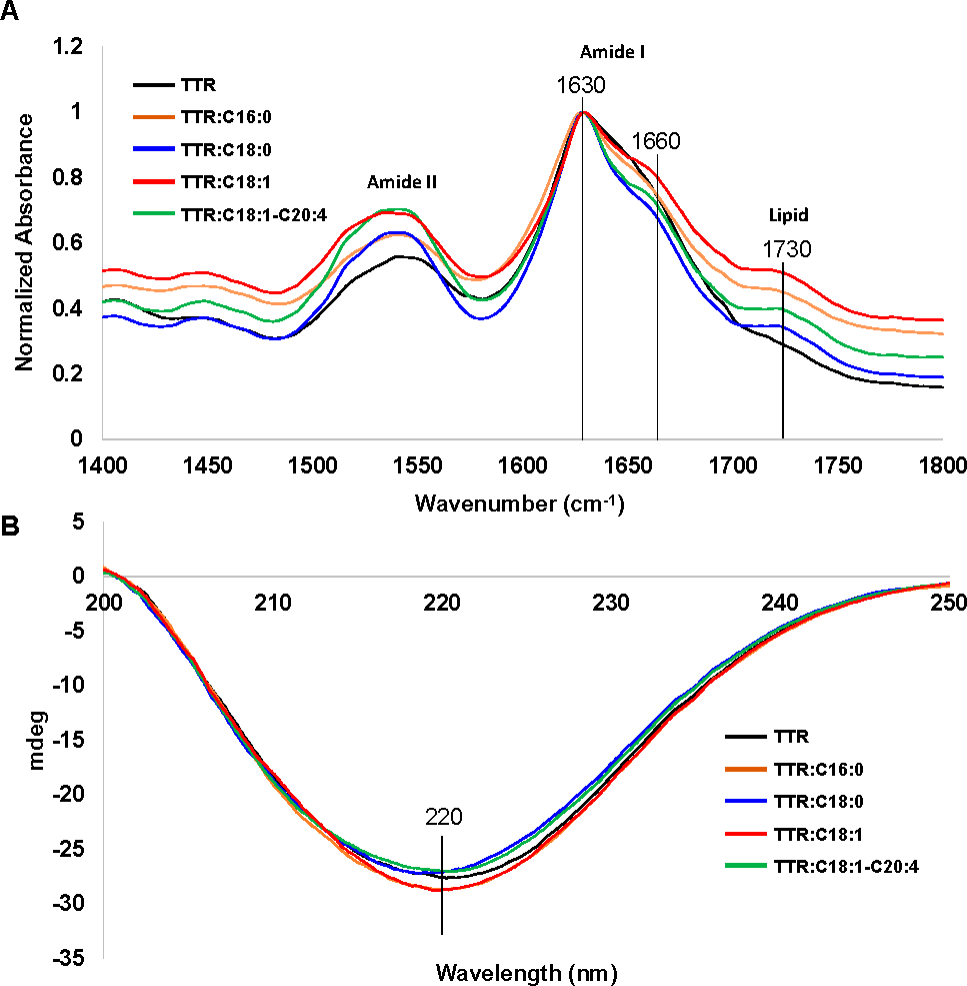
Examination of the secondary structure of the TTR aggregates formed in the presence and absence of CPs. IR (A) and CD (B) spectra acquired from TTR and TTR:C16:0, TTR:C18:0, TTR:C18:1, and TTR:C18:1-C20:4.

IR spectra acquired from all samples exhibited amide I at ∼1630 cm^–1^, which indicated the predominance of parallel β-sheet in the secondary structure of TTR fibrils formed in the presence and absence of CPs, Figure 3A. These results indicated that the secondary structure of the analyzed protein samples was very similar. This conclusion is further supported by CD results, Figure 3B. We found that CD spectra acquired from all samples exhibited a negative peak at ∼220 nm. This spectroscopic signature indicates the predominance of the β-sheet in the secondary structure in the TTR fibrils formed in both the presence and absence of CPs. In the acquired IR spectra, we also observed a shoulder at 1660 cm^–1^, which could be assigned to unordered protein secondary structure. Thus, we can conclude that all analyzed samples had a small amount of unaggregated TTR. It should be noted that IR spectra acquired from TTR:C16:0, TTR:C18:0, TTR:C18:1, and TTR:C18:1-C20:4 CPs had a vibrational band at 1730 cm^–1^, which can be assigned to the carbonyl (C=O) vibration of CPs.^25^ As expected, this vibrational band was absent in the IR spectrum of the TTR.

In our previous studies, we demonstrated that, although conventional FTIR spectroscopy could be used to probe the aggregation state of amyloid proteins, it did not allow for elucidation of the secondary structure of individual amyloid oligomers and fibrils.^19,21,25^ To overcome this limitation, atomic force microscopy Infrared (AFM-IR) spectroscopy can be employed.^26−29^ AFM-IR allows for a precise positioning of the metallized scanning AFM tip directly at the object of interest.^30−32^ Next, pulsed tunable IR light is used to create thermal vibrations in the sample.^33,34^ These vibrations are passed to the scanning probe and converted into IR spectra.^35−37^

Using AFM-IR, we were able to resolve the secondary structure of individual fibrils observed in TTR and TTR:C16:0, TTR:C18:0, TTR:C18:1, and TTR:C18:1-C20:4, Figure 4 and Figures S1–S10.

**Figure 4:**
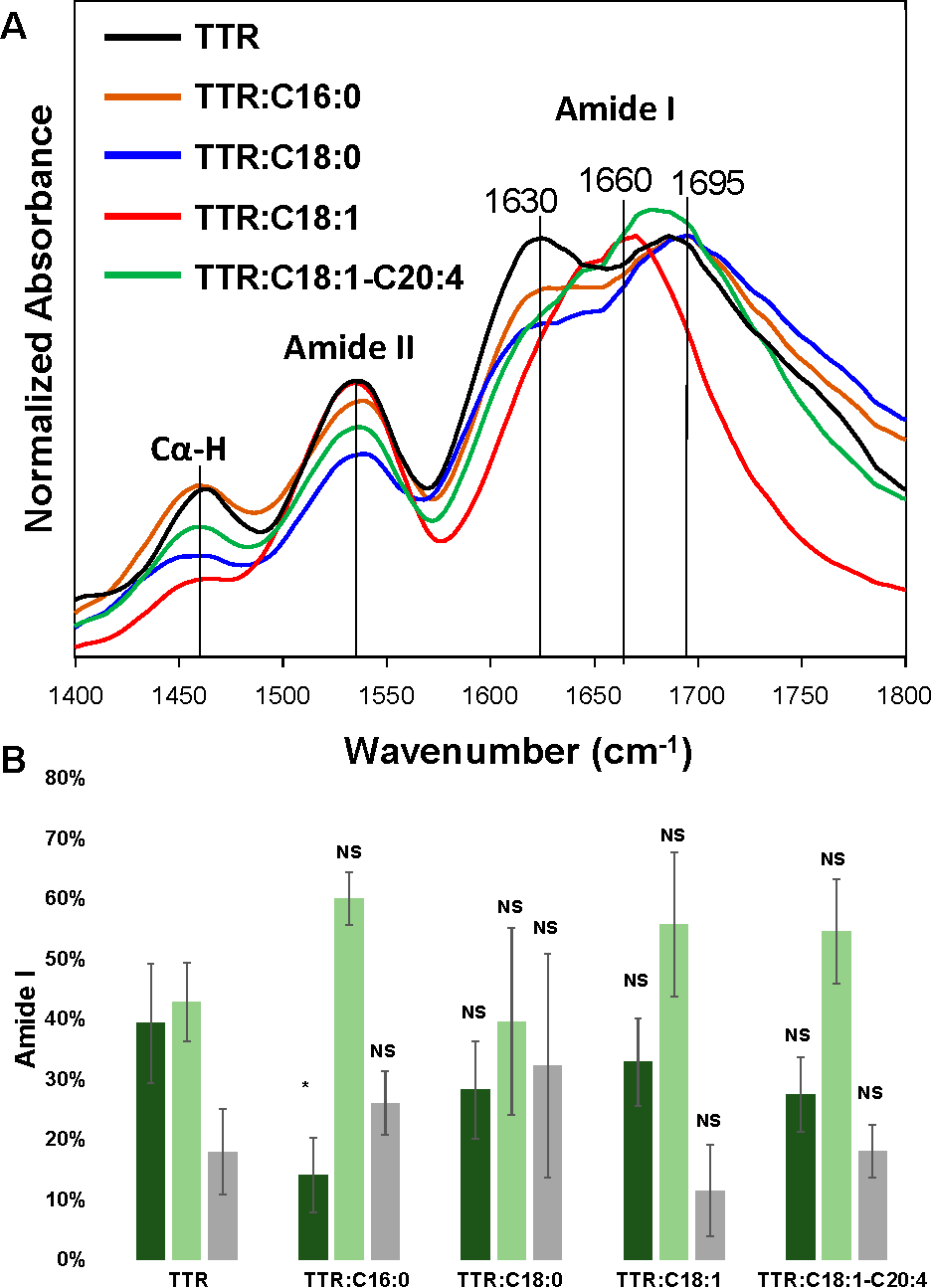
Averaged AFM-IR spectra (A) acquired from TTR and TTR:C16:0, TTR:C18:0, TTR:C18:1, and TTR:C18:1-C20:4 fibrils. A bar graph (B) summarizes the distribution of protein secondary structure in the protein aggregates according to the fitting of the amide I band. Parallel β-sheet (1630 cm^–1^) in green, α-helix and random coil (1660 cm^–1^) in light green, and antiparallel β-sheet (1695 cm^–1^) in gray. AFM-IR spectra were acquired using a nanoIR3 system equipped with a QCL laser.

AFM-IR revealed high similarities in the secondary structures of TTR and TTR:C16:0, TTR:C18:0, TTR:C18:1, and TTR:C18:1-C20:4 fibrils. However, we found a significantly lower amount of parallel β-sheet in the structure of TTR:C16:0 fibrils compared to that of TTR aggregated formed in the lipid-free environment. Based on these results, we can conclude that the presence of C16:0 CPs lowered the amount of parallel β-sheets in the structure of TTR fibrils. We also observed higher amounts of α-helix and random coil in the secondary structure of TTR:C16:0, TTR:C:18:1, and TTR:C18:1-C20:4 fibrils compared to TTR fibrils formed in the lipid-free environment. These results indicate that the presence of CPs increased the amount of α-helix and random coil in the secondary structure of TTR fibrils that were formed in the presence of CPs. We found large deviations in the secondary structure of TTR:C18:0 fibrils that were analyzed by AFM-IR. It should be noted that CPs themselves do not exhibit intense vibrational bands in the amide I region (Figure S11). Elucidation of structural diversity of these aggregates is a subject for a separate study that is out of the scope of the current work.^26^

Finally, we tested cytotoxicity of TTR aggregates formed in the presence and absence of CPs using a rat N27 dopaminergic cell line, Figure 5.

**Figure 5:**
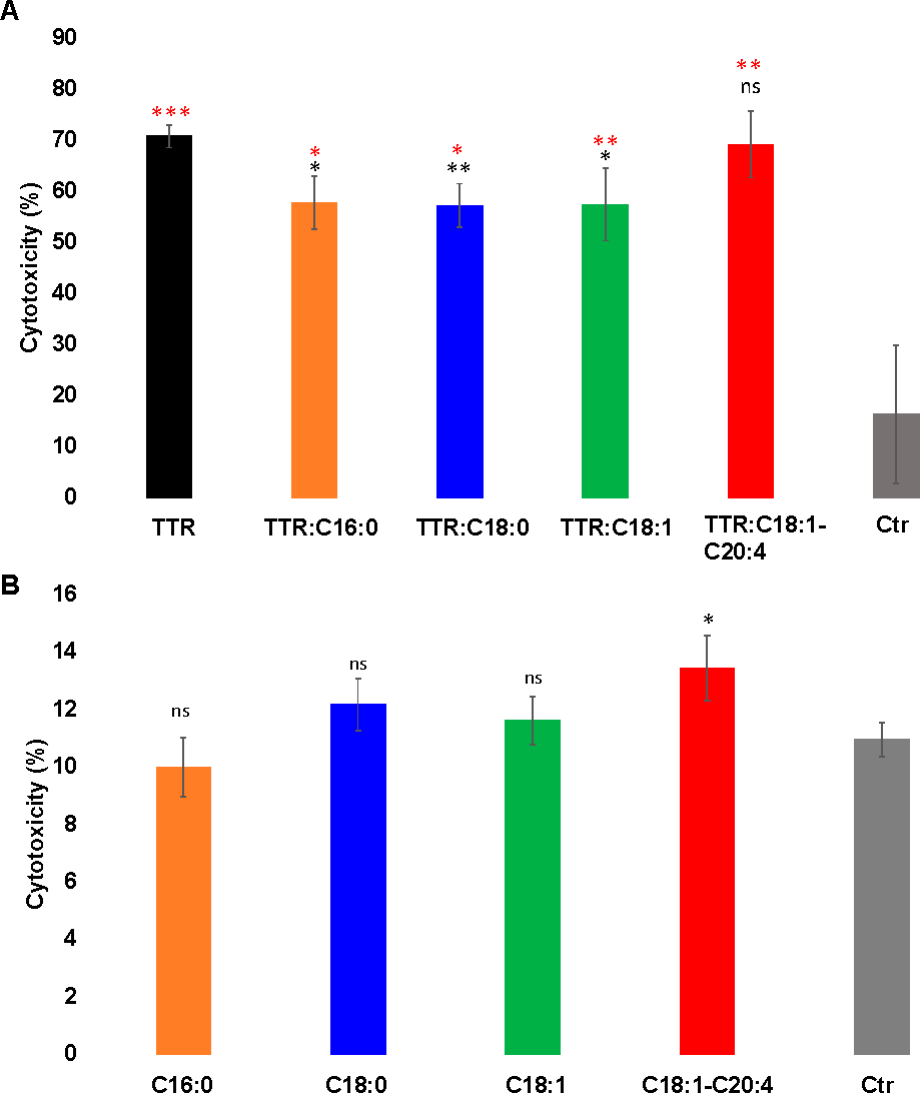
Cytotoxicity of TTR aggregates formed in the presence and absence of CPs. Results of LDH assay (*n* = 3) indicate toxicity of TTR and TTR:C16:0, TTR:C18:0, TTR:C18:1, and TTR:C18:1-C20:4 (A), as well as toxicity of CPs themselves (B) to rat N27 dopaminergic cells. *p < 0.05, **p < 0.01, ***p < 0.001. NS- nonsignificant difference.

We found that all protein aggregates exerted significant cell toxicity compared with the control. At the same time, we found that TTR:C16:0, TTR:C18:0, and TTR:C18:1 were significantly less toxic to N27 cells than TTR fibrils formed in the CPs-free environment, Figure 5. We also found that the presence of C18:1-C20:4 did not change the toxicity of TTR aggregates (TTR:C18:1-C20:4) compared to TTR fibrils (TTR) formed in the absence of CPs. Finally, we found that all CPs themselves, except C18:1-C20:4, did not exert any significant toxicity to N27 cells. It should be noted that C18:1-C20:4 was found to be more toxic to the cells compared with the control. These findings show that C16:0, C18:0 and C18:1 reduce cytotoxicity of TTR fibrils, Figure 5.

Our results demonstrated that an equimolar concentration of CP-SUVs substantially decelerated TTR aggregation when present at the first stages of aggregation. One may expect that CPs stabilized monomeric TTR, inhibiting its tendency to aggregate. Esbjörner’s group previously found such phospholipid-induced stabilization for amyloid β_1–42_ (Aβ_1–42_).^38^ The researchers found that lipid membranes stabilized Aβ_1–42_ and reduced the aggregation rate. Our findings showed that the stabilization effect directly depended on the saturation of FAs in CPs. TTR exhibited much greater stability in the presence of CPs with unsaturated FAs compared to CPs with saturated FAs. Similar findings were reported by Ali and co-workers for PS. It was found that unsaturated 1,2-dioleoyl-*sn*-glycero-3-phospho-l-serine with two double bounds (18:1, DOPS) decelerated TTR aggregation much greater than fully saturated 1,2-dimyristoyl-*sn*-glycero-3-phospho-l-serine (14:0, DMPS) and 1,2-distearyl-*sn*-glycero3-phospho-l-serine (18:0, DSPS), as well as PS with one double bound, 1-palmitoyl-2-oleoyl-*sn*-glycero-3-phospho-l-serine (16:0–18:1, POPS).^21^ It should be noted that a completely opposite effect of PS with different length and saturation of FAs was recently reported by Ali and co-workers for wild-type α-synuclein.^39^ Specifically, all PSs strongly accelerated rather than decelerated protein aggregation. Thus, we can conclude that the effect of the saturation of FAs in phospholipids on the rate of protein aggregation was directly dependent on the secondary structure of amyloidogenic proteins.

We also found that the saturation of FAs uniquely altered the morphology of TTR aggregates that were grown in the solution that contained CPs. Specifically, much thicker fibrils formed by TTR in the presence of CPs with unsaturated FAs compared with CPs that had fully saturated FAs. Similar effects on the morphology of the protein aggregates were previously reported by Ali and co-workers for PS with different lengths and saturations of FAs, as well as for polyunsaturated FAs.^19−21^ Thus, we can conclude that the FA-determined changes in the morphology of protein aggregates can be a general phenomenon attributed to a large group of phospholipids and polyunsaturated FAs.

Ramamoorthy’s group previously reported that lipid vesicles could lower the toxicity of fibrils formed by Aβ_1–42_ peptide.^40^ Namely, Korshavn et al. showed that Aβ_1–42_ fibrils grown in the presence of LUVs formed by 1,2-dilauroyl-*sn*-glycero-3-phosphatidylcholine (DLPC), a lipid that had short-chain FAs, were less toxic compared to Aβ_1–42_ fibrils formed in the LUV-free environment.^40^ This effect was explained by the LUV-based stabilization of amyloid aggregates. Our results concur with the previous studies by Ramamoorthy’s group. It should be noted that previously Zhaliazka and co-workers found a direct relationship between the amount of parallel β-sheet and toxicity of Aβ_1–42_ fibrils.^41^ Therefore, we infer that the decrease in the toxicity of TTR fibrils could be caused by a decrease in the amount of β-sheets in these aggregates, as was revealed by AFM-IR for TTR:C16:0 fibrils.

Overall, the conclusions drawn from this study are consistent with previous work relating to TTR aggregation in the presence of lipids.^4−6^ With the specific effects of CPs characterized, it would be valuable to investigate the integration of other membrane components with plasmalogens. Previous studies have indicated other phospholipid species and cholesterol are integral in the aggregation and cytotoxicity of TTR.^42^ Thus, the composite effects of multicompound lipid vesicles stand pertinent in the understanding of ATTR and related diseases. It has been reported that reduced plasmalogen levels were consistent among subjects with more prominent signs of neurodegeneration.^43^ In the same effects, plasmalogen dietary supplementation was observed to reduce neurodegeneration in mouse models.^44^ Thus, it is critically important to utilize animal models to examine the relationship between change in the plasmalogen levels and the onset of ATTR.

It should be noted that, in the current study, we examined the secondary structure of mature TTR fibrils. Additional studies are required to reveal the extent to which CPs alter the TTR assembly at the early stages of protein aggregation. In our previous study, we showed that, in the absence of CPs, TTR formed two types of structurally different oligomers.^26^ The first type was observed at ∼3 h. These aggregates persisted during the entire course of protein aggregation. The second type appeared in later stages and instantaneously propagated into the fibrils. Considering these observations, it becomes important to determine whether the same types of oligomers could be formed by TTR in the presence of CPs.

Our results demonstrate that CPs with both saturated and unsaturated FAs strongly suppressed TTR aggregation. We found that CPs with unsaturated FAs exerted a stronger suppression effect compared to CPs with saturated FAs. We also found that CPs with saturated FAs did not change the morphology of the TTR fibrils. At the same time, AFM imaging revealed the presence of much thicker fibrillar species in TTR:C18:1 and TTR:C18:1-C20:4. Although IR and CD did not reveal substantial structural differences between all examined protein aggregates, we found that CPs with C16:0, C18:0, and C18:1 FAs substantially lowered the cytotoxicity of TTR fibrils that were formed in their presence.

## Supporting Information Available

Materials and methods, fitted AFM-IR spectra (Figures S1–S5), representative AFM images that indicate locations from which AFM-IR spectra (Figures S6–S10); IR spectra of SUVs of C16:0, C18:0, C18:1, and C18:1-C20:4 (Figure S11) (PDF)
