# Supplementary material for: Plasmalogens Alter the Aggregation Rate of Transthyretin and Lower Toxicity of Transthyretin Fibrils
Source: J Phys Chem Lett. 2024 Apr 25;15(17):4761–6. doi: 10.1021/acs.jpclett.4c00868 (PMC11071038; doi:10.1021/acs.jpclett.4c00868)
Supplement: Supplementary file 1 — jz4c00868_si_001.pdf [file jz4c00868_si_001.pdf]

Plasmalogens Alter the Aggregation Rate of Transthyretin and Lower Toxicity of Transthyretin Fibrils

Jadon Sitton[1], Abid Ali[1], Luke Osborne[1], Aidan P. Holman[2], Axell Rodriguez[1], and Dmitry Kurouski[1,3*]

1. Department of Biochemistry and Biophysics, Texas A&M University, College Station, Texas 77843, United States
2. Department of Entomology, Texas A&M University, College Station, Texas 77843, United States
3. Department of Biomedical Engineering, Texas A&M University, College Station, Texas, 77843, United States

Email: dkurouski@tamu.edu

Supporting Information

Materials and Methods:

Materials: CPs were purchased from Avanti (Alabaster, AL, USA). TTR was expressed in the lab in *Escherichia coli* Rosetta strain using LB broth media according to the protocol previously reported by our group.[19-21]

Liposome preparation: SUVs of CPs were prepared according to the procedure reported by Mateyenka and co-workers.[45-47] Briefly, lipids were first dissolved in PBS, pH 7.4. Next, a heating-thawing cycle was performed to the lipid solutions. For this, solutions were first heated to 50°C for 30 min and immersed into lipid nitrogen for 3-5 min. Finally, lipid solutions were exposed to the extruding procedure in which the solutions were passed 20-30 times through 100 nm membrane (Avanti, Alabaster, AL, USA). LUV sizes were determined by dynamic light scattering. All samples had LUV sizes of 62.7 nm (C16:0), 42.0 (C18:0), 71.0 (C18:1), and 47.1 nm (C18:1-C20:4).

Protein aggregation and kinetic measurements: 50 μM of TTR was dissolved in 0.1M sodium acetate buffer that contained 1M KCl; pH 3.0. For **protein:lipid samples,** 50 μM of TTR was mixed with an equivalent concentration of the corresponding LUVs. Solution pH was adjusted to pH 3.0. All samples were placed into a well-plate that was agitated for 48 h with 510 rpm, 37 °C (Tecan, Mannedorf, Switzerland). For kinetic measurements, ThT was added to the sample to reach the final concentration of 25 μM. Samples were incubated at the same experimental conditions using the same equipment (Tecan, Mannedorf, Switzerland). Fluorescence measurements were taken every 10 min; excitation was 450 nm; emission was collected at 488 nm.

AFM imaging: AFM images were collected using AIST-NT-HORIBA system (Edison, NJ) in tapping mode. For each measurement, an aliquot of samples incubated at 37 °C for 48h was diluted with PBS and then deposited onto a pre-cleaned glass coverslip. The same was left drying on the glass coverslip surface. After the sample was fully dried, the glass surface was rinsed by DI water and dried under a flow of dry nitrogen. AIST-NT image processing software was used to analyze AFM images. The following image processing steps were made: Facet leveling; Iterative Polynomial Background Leveling; Fit Line Correction; Remove Scar Line Correction.

Circular Dichroism (CD): After 48 h of TTR incubation at 37 °C, protein samples were diluted using PBS and placed into a quartz cuvette. CD spectra were measured immediately using Jasco J1000 CD spectrometer (Jasco, Easton, MD, USA). In total, 3 spectra were collected from each sample from 190 to 240 nm and then averaged.

Attenuated total reflectance Fourier-transform Infrared (ATR-FTIR) spectroscopy: After 48 h of TTR aggregation at 37 °C, protein samples were deposited onto the crystal of 100 FTIR spectrometer (Perkin-Elmer, Waltham, MA, USA) equipped with the ATR module. Samples were dried at room temperature; 3 spectra were collected from each sample and averaged.

AFM-IR: Protein samples were deposited onto a 70 nm gold-coated silicon wafer at a volume of 3-6 µL. The deposited sample was left to dry at room temperature for 15-20 minutes, then rinsed with DI water, and lastly dried with a N_2 air flow. AFM-IR imaging and spectral acquisition was acquired by using a nanoIR3 system (Bruker, Santa Barbara, CA, USA), the source of the IR laser is from a QCL laser. AFM imaging was collected through contact mode using AFM tips (ContGB-G AFM probe, NanoAndMore). The contact-mode tip was optimized using a polymethyl acrylate standard sample for the following wavenumbers: 1400-1800 cm^{-1}. Images were taken at a scan rate of 0.8 Hz with a height and width ranging from 1-10 µm, resolution of 256 for both X and Y parameters, and an I and P gain of 2 and 4 respectively. Laser parameters include a starting power of 25.49%, polarization at 90 deg., a pulse rate around 870 kHz, and an IR focus at 70136 pt. A total of 30 spectra were collected per sample with a co-average of 3 for each spectrum acquired. The spectra were zapped at the 1648-1652 range to remove the artifact caused by the chip-to-chip transition of the instrument. The spectra resolution is 2 cm^{-1}/pt. The spectra were processed by applying a smoothing of Savitzky-Golay at a polynomial order of 0, using MATLAB as the programming language application.

Spectral analysis: Fitting of AFM-IR spectra was performed in GRAMS/AI™ Spectroscopy Software. After amide I region (1570-1800 cm^{-1}) was baselined, automated peak identification was performed. Next, fitting was optimized to reach the best possible overlap of the fitted and experimental spectra. Finally, peak areas were determined. Parallel β-sheet was considered from 1616-1630 cm^{-1}; α-helix and random coil from 1640-1670 cm^{-1}, and anti-parallel β-sheet from 1690-1700 cm^{-1}. All other peaks were discarded from the quantification of the secondary structures. Such peaks could correspond to side chain vibrations (~1600 cm^{-1}) and lipids (1710-1740 cm^{-1}).[1-3]

Cell toxicity assays: The N27 rat dopaminergic neuron cell line was purchased from Sigma-Aldrich (St. Louis, MO). Cells were cultured in 96-well plates with RPMI 1640 Medium supplemented with 10% fetal bovine serum (FBS) at 37°C with 5% CO_2. Once the cells reached ~70% confluency after 24 hours, they were used for subsequent experiments. For the LDH assay, 100 µL of the cell culture was replaced with 100 µL of RPMI 1640 Medium containing 5% FBS and 10 µL of the protein samples. After 24 hours of incubation, the amount of lactate dehydrogenase (LDH) released into the cell culture medium was measured using the non-radioactive CytoTox 96 cytotoxicity assay kit (G1781, Promega, Madison, WI, USA). The toxicity of the protein aggregates towards N27 cells was measured by the level of formazan produced, which directly correlated with the amount of LDH released using absorbance read at 490 nm.

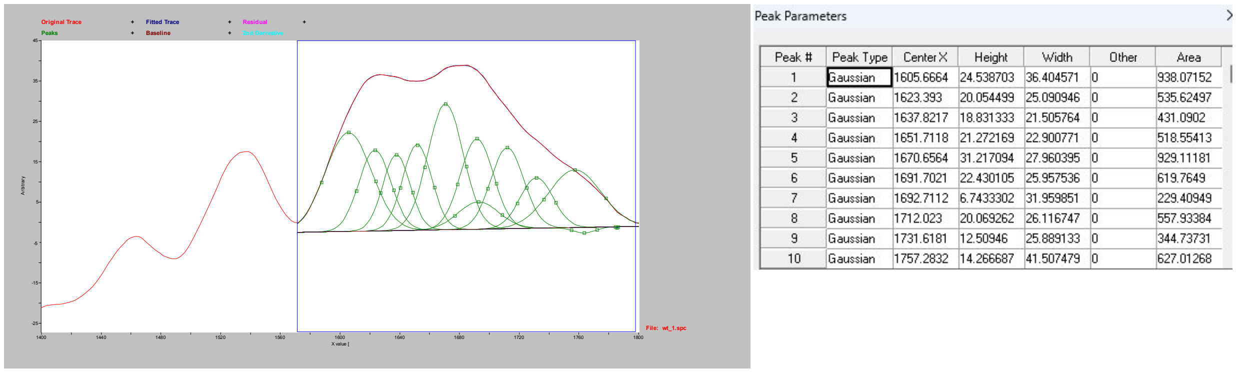

Peak Parameters

Peak #	Peak Type	Center X	Height	Width	Other	Area
1	Gaussian	1605.6664	24.538703	36.404571	0	938.07152
2	Gaussian	1623.393	20.054499	25.090946	0	535.62497
3	Gaussian	1637.8217	18.831333	21.505764	0	431.0902
4	Gaussian	1651.7118	21.272169	22.900771	0	518.55413
5	Gaussian	1670.6564	31.217094	27.960395	0	929.11181
6	Gaussian	1691.7021	22.430105	25.957536	0	619.7649
7	Gaussian	1692.7112	6.7433302	31.959851	0	229.40949
8	Gaussian	1712.023	20.069262	26.116747	0	557.93384
9	Gaussian	1731.6181	12.50946	25.889133	0	344.73731
10	Gaussian	1757.2832	14.266687	41.507479	0	627.01268

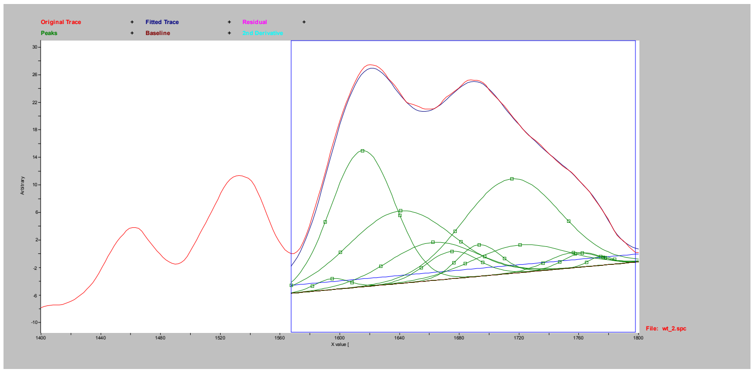

Peak Parameters

Peak #	Peak Type	Center X	Height	Width	Other	Area
1	Gaussian	1567.3469	1.1627008	2.46E+19	0	272.87877
2	Gaussian	1594.7895	1.5934289	26.617099	0	44.797577
3	Gaussian	1615.1173	19.786403	50.047031	0	1041.0683
4	Gaussian	1640.5705	10.548478	80.806251	0	892.40384
5	Gaussian	1662.1303	5.5372889	69.417243	0	408.8952
6	Gaussian	1675.0374	3.9947847	41.242817	0	175.37751
7	Gaussian	1693.1261	4.5913469	33.810467	0	165.24316
8	Gaussian	1715.046	13.707598	75.932861	0	1104.1467
9	Gaussian	1720.7028	4.0235881	73.667348	0	314.06633
10	Gaussian	1756.1519	2.1963664	40.311509	0	93.919737

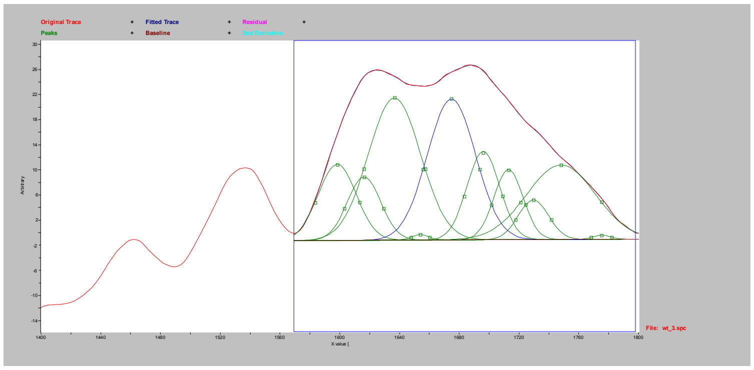

Peak Parameters

Peak #	Peak Type	Center X	Height	Width	Other	Area
1	Gaussian	1598.4272	12.122875	29.914767	0	381.67834
2	Gaussian	1616.3217	10.074739	26.347411	0	282.55155
3	Gaussian	1636.69	22.670076	41.135688	0	992.60879
4	Gaussian	1653.9334	.88912083	12.503727	0	11.834025
5	Gaussian	1674.8119	22.469885	37.964406	0	908.04979
6	Gaussian	1696.1396	14.039477	25.842469	0	386.20434
7	Gaussian	1713.0143	11.149448	22.964501	0	272.54774
8	Gaussian	1729.638	6.3370763	24.012516	0	161.97904
9	Gaussian	1748.1362	11.860819	54.079094	0	676.46406
10	Gaussian	1775.1934	.64448377	13.771282	0	9.447575

Figure S1 Fitted AFM-IR spectra with the corresponding peak areas for TTR. Each averaged spectrum corresponds to 10 spectra acquired from different fibrils.

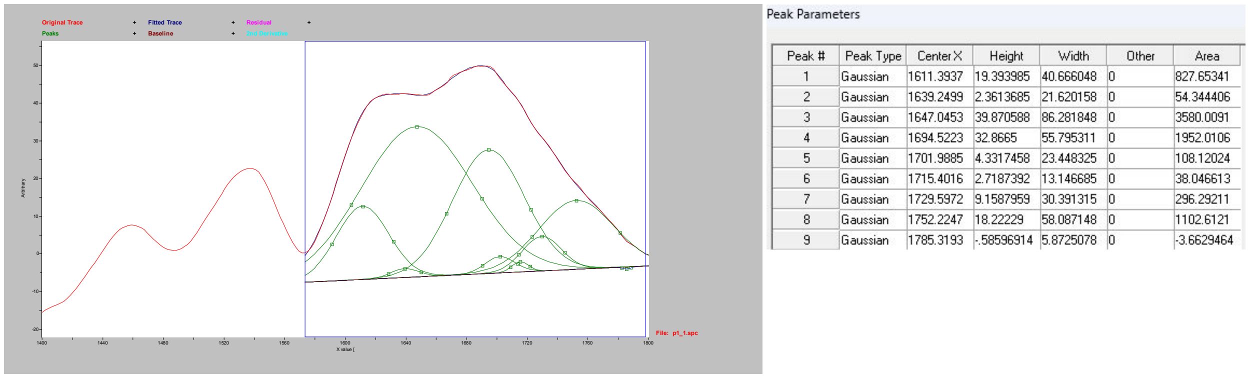

Peak Parameters

Peak #	Peak Type	Center X	Height	Width	Other	Area
1	Gaussian	1611.3937	19.393985	40.666048	0	827.65341
2	Gaussian	1639.2499	2.3613685	21.620158	0	54.344406
3	Gaussian	1647.0453	39.870588	86.281848	0	3580.0091
4	Gaussian	1694.5223	32.8665	55.795311	0	1952.0106
5	Gaussian	1701.9885	4.3317458	23.448325	0	108.12024
6	Gaussian	1715.4016	2.7187392	13.146685	0	38.046613
7	Gaussian	1729.5972	9.1587959	30.391315	0	296.29211
8	Gaussian	1752.2247	18.22229	58.087148	0	1102.6121
9	Gaussian	1785.3193	-.58596914	5.8725078	0	-3.6629464

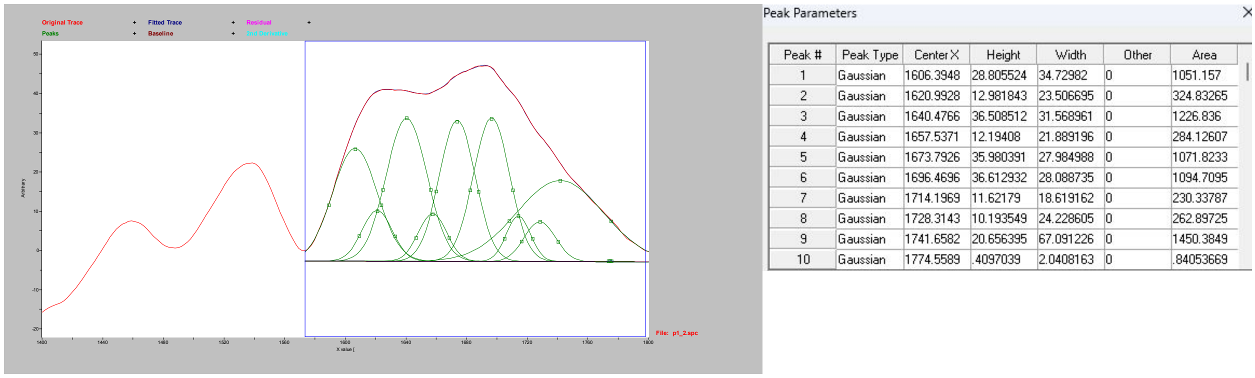

Peak Parameters

Peak #	Peak Type	Center X	Height	Width	Other	Area
1	Gaussian	1606.3948	28.805524	34.72982	0	1051.157
2	Gaussian	1620.9928	12.981843	23.506695	0	324.83265
3	Gaussian	1640.4766	36.508512	31.568961	0	1226.836
4	Gaussian	1657.5371	12.19408	21.889196	0	284.12607
5	Gaussian	1673.7926	35.980391	27.984988	0	1071.8233
6	Gaussian	1696.4696	36.612932	28.088735	0	1094.7095
7	Gaussian	1714.1969	11.62179	18.619162	0	230.33787
8	Gaussian	1728.3143	10.193549	24.228605	0	262.89725
9	Gaussian	1741.6582	20.656395	67.091226	0	1450.3849
10	Gaussian	1774.5589	.4097039	2.0408163	0	.84053669

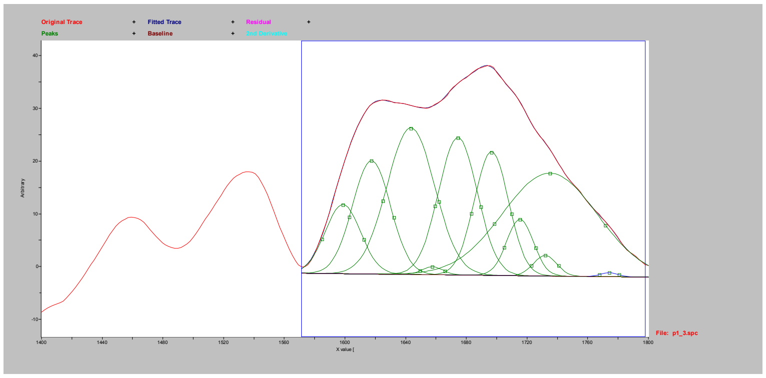

Peak Parameters

Peak #	Peak Type	Center X	Height	Width	Other	Area
1	Gaussian	1598.7224	13.090426	27.459016	0	378.88131
2	Gaussian	1617.5418	21.491987	29.268038	0	669.50823
3	Gaussian	1643.4608	27.797687	36.935263	0	1092.9017
4	Gaussian	1657.6688	1.5119559	16.295199	0	26.225935
5	Gaussian	1674.4907	26.024685	30.060789	0	832.75656
6	Gaussian	1696.5904	23.446157	26.477783	0	660.82352
7	Gaussian	1715.3718	10.68825	20.635893	0	234.78053
8	Gaussian	1731.9144	3.9257871	17.753794	0	74.190813
9	Gaussian	1735.1083	19.483885	73.248191	0	1495.5486
10	Gaussian	1774.3096	.77830431	13.024369	0	10.790429

Figure S2. Fitted AFM-IR spectra with the corresponding peak areas for TTR:C16:0. Each averaged spectrum corresponds to 10 spectra acquired from different fibrils.

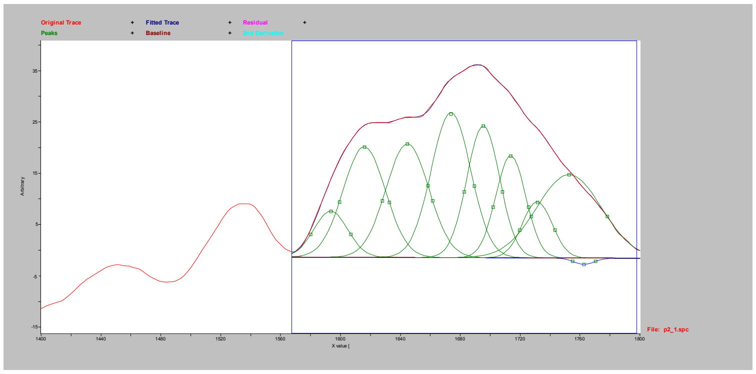

Peak Parameters

Peak #	Peak Type	Center X	Height	Width	Other	Area
1	Gaussian	1593.3937	8.9678973	26.707755	0	252.14874
2	Gaussian	1615.7612	21.545682	33.330012	0	764.1687
3	Gaussian	1644.6437	22.145622	33.690591	0	794.19786
4	Gaussian	1673.7232	28.317797	30.964065	0	933.36101
5	Gaussian	1695.3195	25.743043	25.734701	0	705.19824
6	Gaussian	1713.6692	19.907108	23.556717	0	499.17768
7	Gaussian	1731.3866	10.972817	23.468711	0	274.11928
8	Gaussian	1752.719	16.283124	50.838672	0	871.58074
9	Gaussian	1762.6047	-1.2077937	15.555236	0	-19.998691

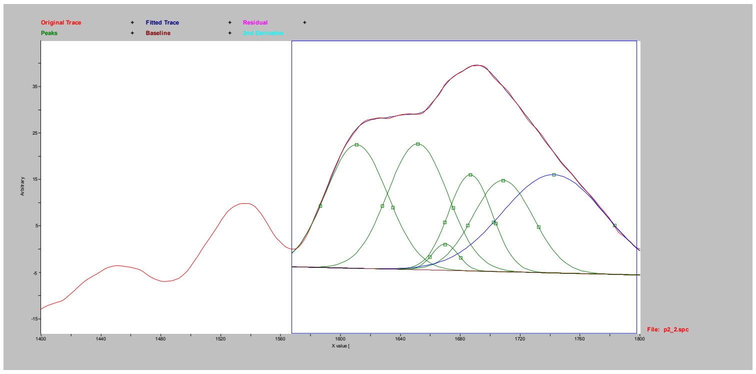

Peak Parameters

Peak #	Peak Type	Center X	Height	Width	Other	Area
1	Gaussian	1610.696	26.659024	48.533431	0	1352.7463
2	Gaussian	1651.5252	27.167266	47.218965	0	1365.4904
3	Gaussian	1669.9135	5.7156287	20.351267	0	123.81911
4	Gaussian	1686.6812	20.778483	34.138012	0	755.06486
5	Gaussian	1708.596	19.672608	47.199097	0	988.38753
6	Gaussian	1742.5325	21.175995	80.905348	0	1748.2032

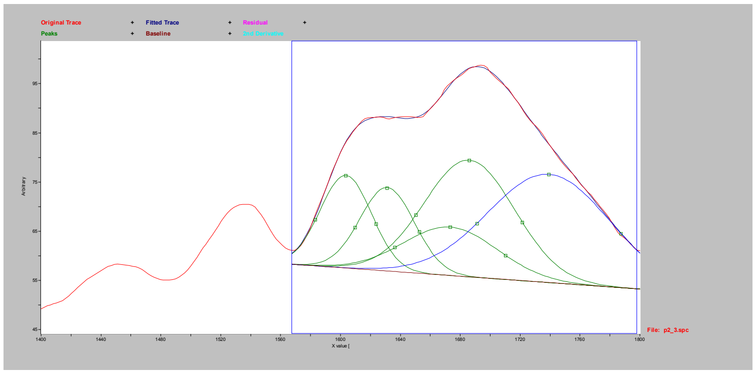

Peak Parameters

Peak #	Peak Type	Center X	Height	Width	Other	Area
1	Gaussian	1603.4677	18.836034	40.530255	0	797.98412
2	Gaussian	1631.1156	16.971761	42.900824	0	774.85841
3	Gaussian	1673.2504	9.849564	73.90761	0	774.5837
4	Gaussian	1685.9369	23.740554	71.104395	0	1796.6996
5	Gaussian	1739.1811	22.017261	96.30229	0	2117.179

Figure S3. Fitted AFM-IR spectra with the corresponding peak areas for TTR:C18:0. Each averaged spectrum corresponds to 10 spectra acquired from different fibrils.

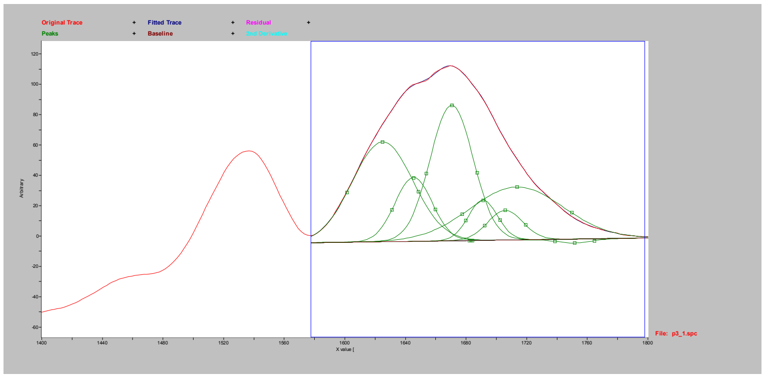

Peak Parameters

Peak #	Peak Type	Center X	Height	Width	Other	Area
1	Gaussian	1624.8665	65.868708	47.145657	0	3275.5185
2	Gaussian	1645.347	42.183265	28.664111	0	1287.0958
3	Gaussian	1670.4495	89.366098	33.357122	0	3173.1718
4	Gaussian	1683.0813	-.014484825	2.0408163	0	-.031019602
5	Gaussian	1691.1305	26.43994	22.69305	0	638.68328
6	Gaussian	1705.8079	19.626942	27.071221	0	565.57824
7	Gaussian	1713.4785	34.880568	72.281031	0	2678.5749
8	Gaussian	1751.4792	-2.5718314	25.990185	0	-71.151421

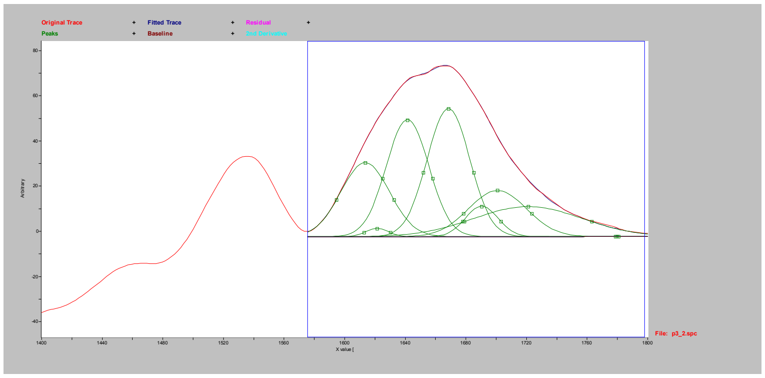

Peak Parameters

Peak #	Peak Type	Center X	Height	Width	Other	Area
1	Gaussian	1613.5158	32.820042	37.845055	0	1310.1217
2	Gaussian	1621.4117	3.6756286	17.49153	0	68.437107
3	Gaussian	1641.5007	51.813646	32.907715	0	1814.9872
4	Gaussian	1668.5165	56.663099	33.126868	0	1998.0802
5	Gaussian	1690.4772	13.440422	25.377155	0	363.06807
6	Gaussian	1700.9115	20.445819	44.850855	0	976.12948
7	Gaussian	1721.016	13.270177	83.951111	0	1172.2944
8	Gaussian	1779.5985	.3440112	2.0408163	0	.78983404

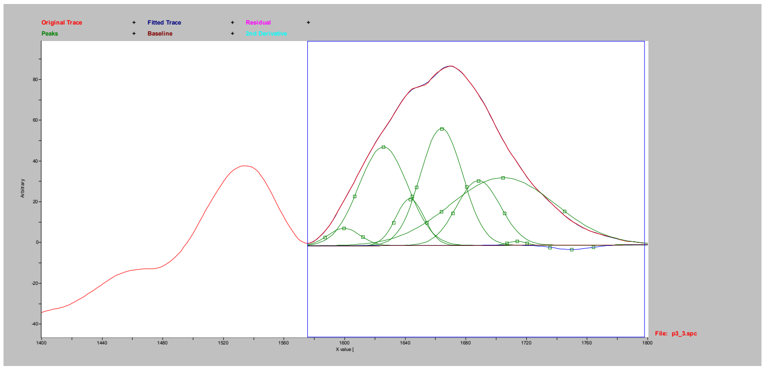

Peak Parameters

Peak #	Peak Type	Center X	Height	Width	Other	Area
1	Gaussian	1599.4625	8.5448105	24.779868	0	222.76804
2	Gaussian	1625.5208	48.510716	37.997364	0	1960.1806
3	Gaussian	1643.1294	22.965916	21.902653	0	535.44233
4	Gaussian	1663.9506	57.52138	32.873963	0	2012.86
5	Gaussian	1688.3855	31.752744	33.981874	0	1148.5788
6	Gaussian	1704.475	33.130876	81.378651	0	2862.9574
7	Gaussian	1713.7397	1.8936951	13.161191	0	26.530013
8	Gaussian	1749.7394	-2.2095135	28.550044	0	-67.148047

Figure S4. Fitted AFM-IR spectra with the corresponding peak areas for TTR:C18:1. Each averaged spectrum corresponds to 10 spectra acquired from different fibrils.

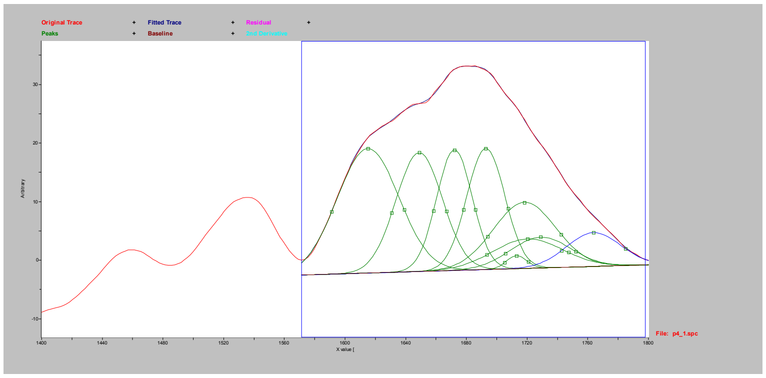

Peak Parameters

Peak #	Peak Type	Center X	Height	Width	Other	Area
1	Gaussian	1615.1419	21.28124	47.821169	0	1066.2379
2	Gaussian	1648.9184	20.307308	36.153498	0	781.51043
3	Gaussian	1672.1519	20.640783	27.649391	0	607.49674
4	Gaussian	1692.8118	20.72981	29.518623	0	651.36382
5	Gaussian	1713.1071	2.2413921	15.568775	0	37.145353
6	Gaussian	1718.2994	11.257881	48.315558	0	578.98462
7	Gaussian	1720.3836	5.0567037	53.823778	0	289.6685
8	Gaussian	1728.9693	5.292104	46.38208	0	261.25789
9	Gaussian	1763.7958	5.7544243	42.016675	0	253.37577

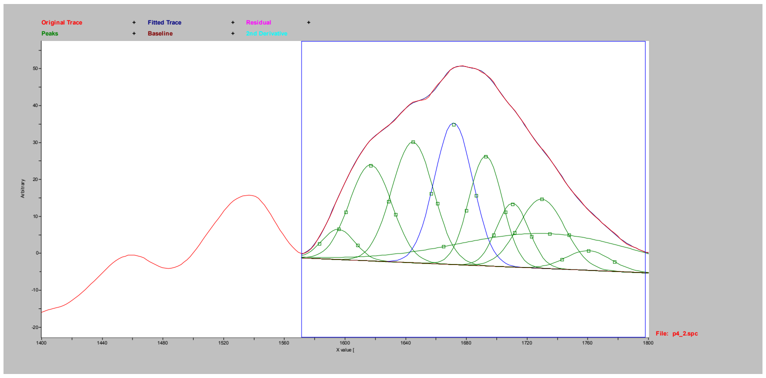

Peak Parameters

Peak #	Peak Type	Center X	Height	Width	Other	Area
1	Gaussian	1595.7213	8.2419331	25.332494	0	219.54205
2	Gaussian	1616.9387	26.02271	32.792233	0	907.85334
3	Gaussian	1644.7113	32.719175	32.33099	0	1126.0393
4	Gaussian	1671.6128	38.385388	29.683555	0	1212.8694
5	Gaussian	1692.792	29.814	25.890379	0	821.65758
6	Gaussian	1710.53	17.312646	24.963318	0	460.04251
7	Gaussian	1729.6961	18.817131	35.695336	0	714.98476
8	Gaussian	1734.8773	9.5244092	139.80573	0	1230.5837
9	Gaussian	1760.401	5.340229	34.650777	0	196.55341

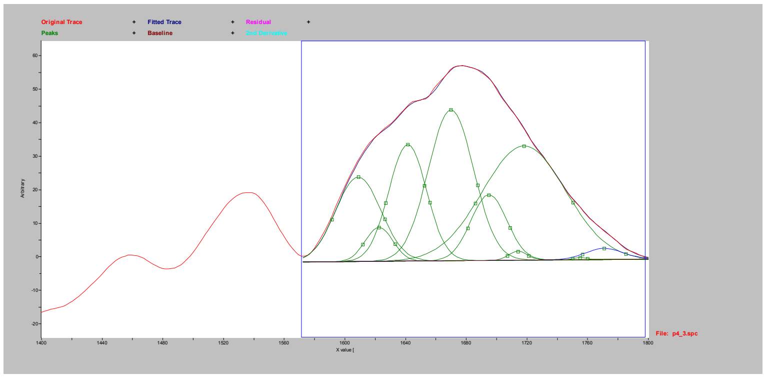

Peak Parameters

Peak #	Peak Type	Center X	Height	Width	Other	Area
1	Gaussian	1608.811	25.21011	34.939702	0	930.92874
2	Gaussian	1622.5012	10.214456	21.333461	0	231.95769
3	Gaussian	1641.3769	34.807778	29.255868	0	1083.9806
4	Gaussian	1669.9029	45.079778	35.11716	0	1685.1298
5	Gaussian	1694.9142	19.622061	28.036066	0	585.59035
6	Gaussian	1714.055	2.6619846	14.19636	0	40.226732
7	Gaussian	1718.0893	34.072351	64.48953	0	2336.4871
8	Gaussian	1754.9748	.63539981	9.7581607	0	6.6000505
9	Gaussian	1770.8913	3.3240463	28.550561	0	100.56107

Figure S5. Fitted AFM-IR spectra with the corresponding peak areas for TTR:C18:1-C20:4. Each averaged spectrum corresponds to 10 spectra acquired from different fibrils.

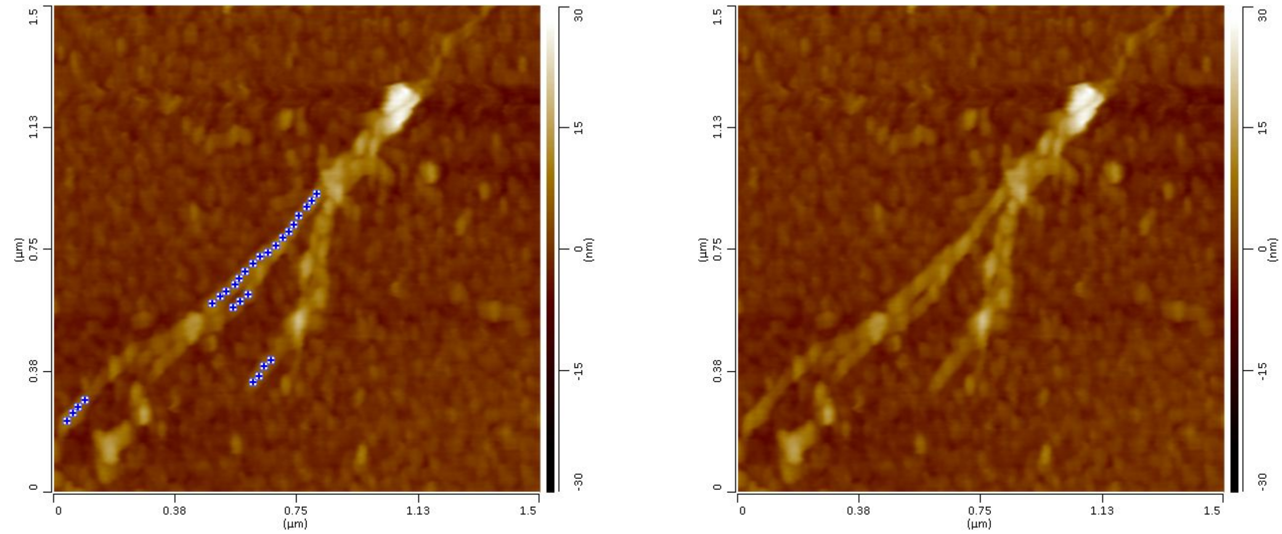

Figure S6. AFM image of TTR:C16:0 fibrils with marked points at which AFM-IR spectra were acquired (left) together with the intact AFM image of these aggregates (right).

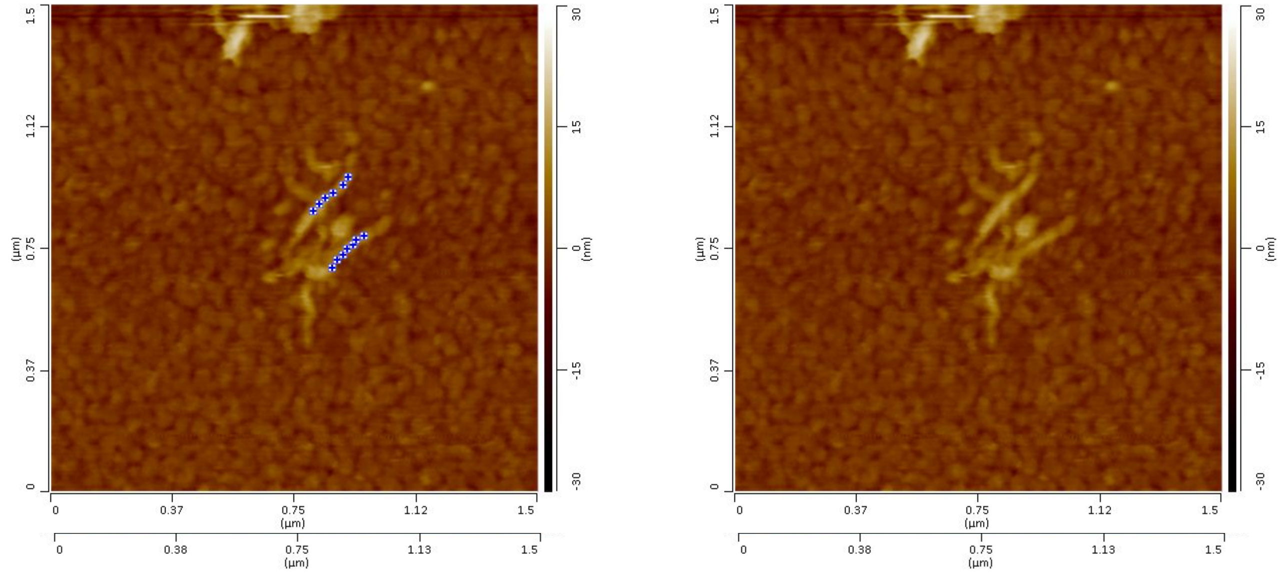

Figure S7. AFM image of TTR:C18:0 fibrils with marked points at which AFM-IR spectra were acquired (left) together with the intact AFM image of these aggregates (right)

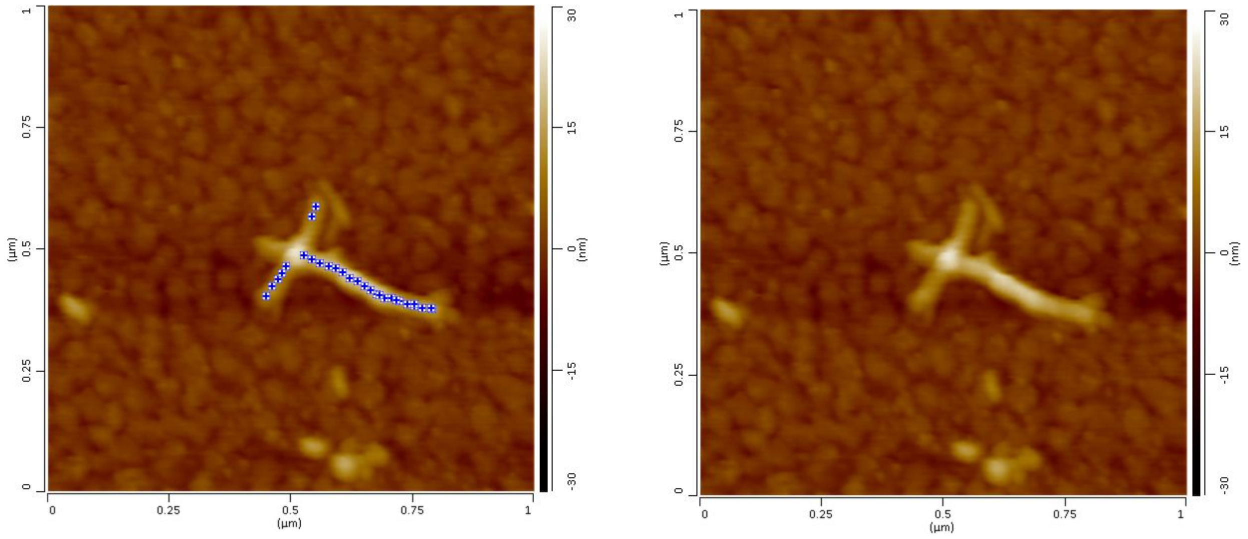

Figure S8. AFM image of TTR:C18:1 fibrils with marked points at which AFM-IR spectra were acquired (left) together with the intact AFM image of these aggregates (right)

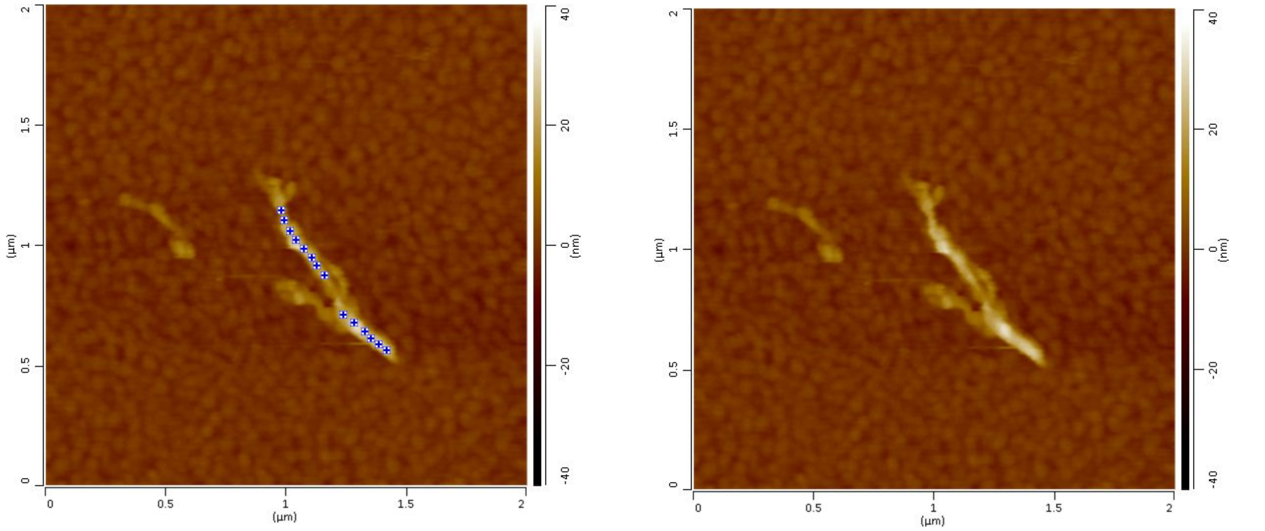

Figure S9. AFM image of TTR:C18:1-C20:4 fibrils with marked points at which AFM-IR spectra were acquired (left) together with the intact AFM image of these aggregates (right).

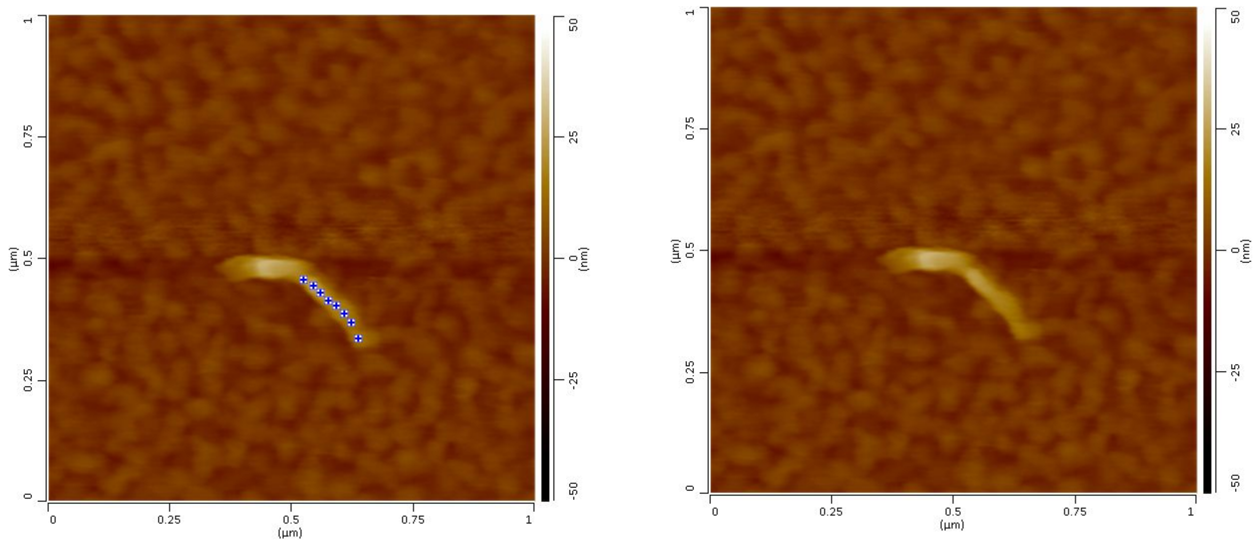

Figure S10. AFM image of TTR fibrils with marked points at which AFM-IR spectra were acquired (left) together with the intact AFM image of these aggregates (right).

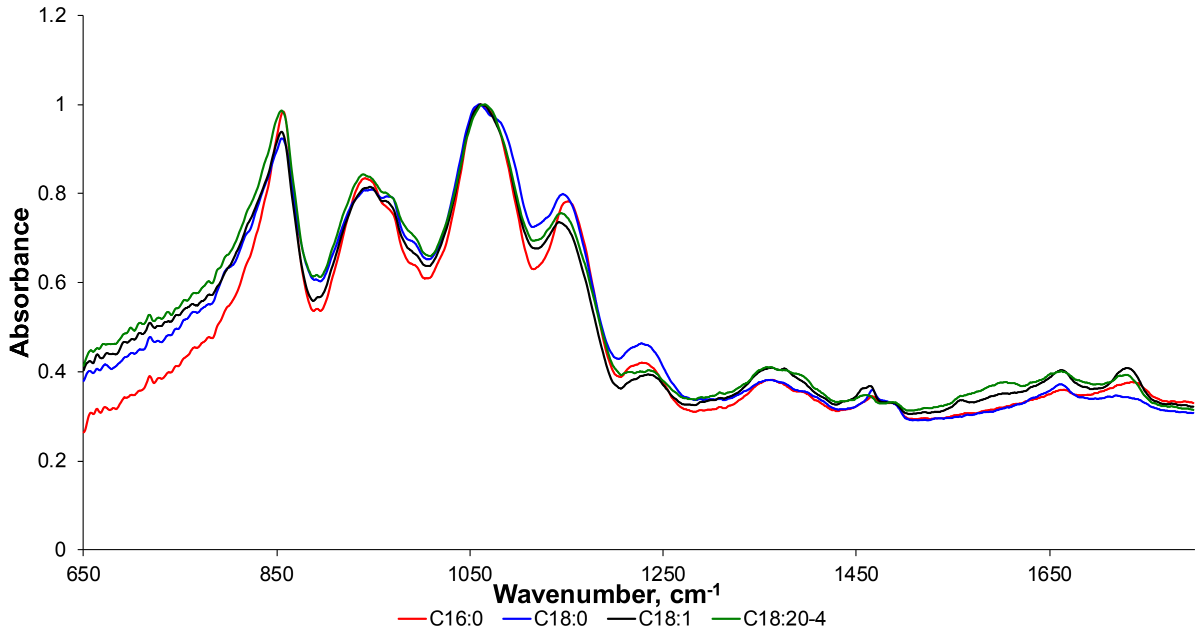

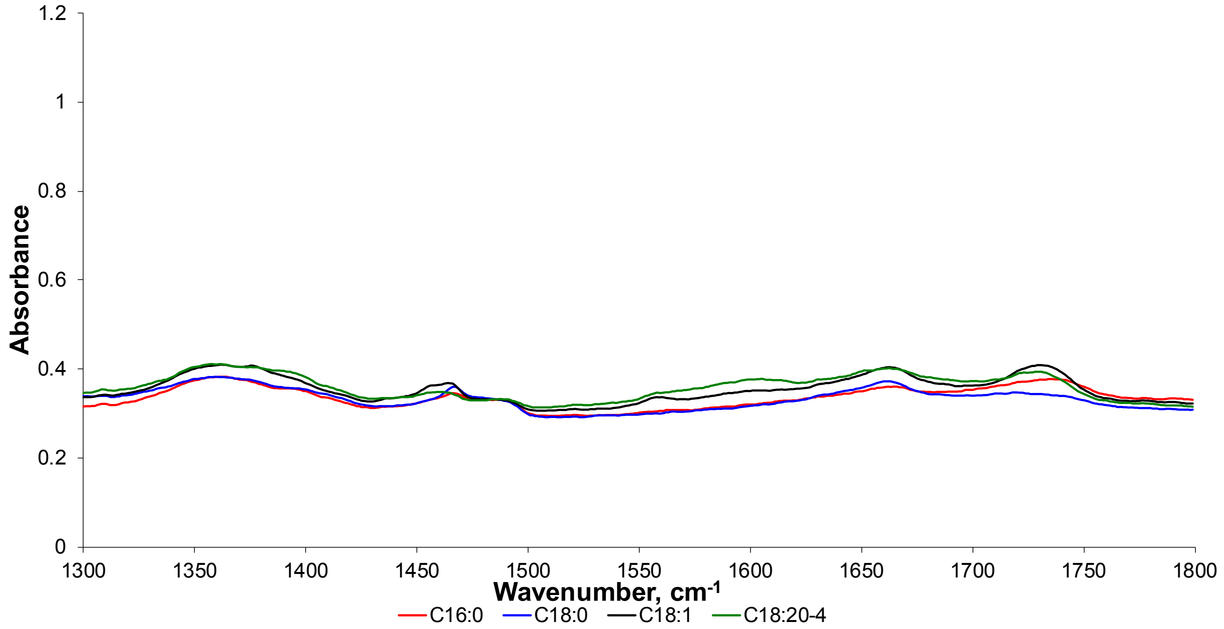

Figure S11. Full (top) and amide I – amide II region (bottom) IR spectra of SUVs of C16:0, C18:0, C18:1, C18:1-C20:4.

(1) Zhaliazka, K.; Kurouski, D. Nanoscale Characterization of Parallel and Antiparallel beta-Sheet Amyloid Beta 1-42 Aggregates. *ACS Chem Neurosci* **2022**, *13* (19), 2813-2820. DOI: 10.1021/acschemneuro.2c00180.
(2) Zhaliazka, K.; Kurouski, D. Nanoscale imaging of individual amyloid aggregates extracted from brains of Alzheimer and Parkinson patients reveals presence of lipids in alpha-synuclein but not in amyloid beta(1-42) fibrils. *Protein Sci* **2023**, *32* (4), e4598. DOI: 10.1002/pro.4598.
(3) Rizevsky, S.; Matveyenka, M.; Kurouski, D. Nanoscale Structural Analysis of a Lipid-Driven Aggregation of Insulin. *J Phys Chem Lett* **2022**, *13* (10), 2467-2473. DOI: 10.1021/acs.jpclett.1c04012.
